# MicroRNA 142-3p Mediates Post-Transcriptional Regulation of D1 Dopamine Receptor Expression

**DOI:** 10.1371/journal.pone.0049288

**Published:** 2012-11-12

**Authors:** Krishna E. Tobón, Denis Chang, Eldo V. Kuzhikandathil

**Affiliations:** Department of Pharmacology and Physiology, University of Medicine and Dentistry of New Jersey-New Jersey Medical School, Newark, New Jersey, United States of America; Wake Forest University, United States of America

## Abstract

The D1 dopamine receptor subtype is expressed in the brain, kidney and lymphocytes. D1 receptor function has been extensively studied and the receptor has been shown to modulate a wide range of physiological functions and behaviors. The expression of D1 receptor is known to change during development, disease states and chronic treatment; however, the molecular mechanisms that mediate the changes in D1 receptor expression under these circumstances are not well understood. While previous studies have identified extracellular factors and signaling mechanisms regulating the transcription of D1 receptor gene, very little is known about other regulatory mechanisms that modulate the expression of the D1 receptor gene. Here we report that the D1 receptor is post-transcriptionally regulated during postnatal mouse brain development and in the mouse CAD catecholaminergic neuronal cell line. We demonstrate that this post-transcriptional regulation is mediated by a molecular mechanism involving noncoding RNA. We show that the 1277 bp 3′untranslated region of D1 receptor mRNA is necessary and sufficient for mediating the post-transcriptional regulation. Using deletion and site-directed mutagenesis approaches, we show that the D1 receptor post-transcriptional regulation is specifically mediated by microRNA miR-142-3p interacting with a single consensus binding site in the 1277 bp 3′untranslated region of D1 receptor mRNA. Inhibiting endogenous miR-142-3p in CAD cells increased endogenous D1 receptor protein expression levels. The increase in D1 receptor protein levels was biologically significant as it resulted in enhanced D1 receptor-mediated signaling, determined by measuring the activation of both, adenylate cyclase and, the dopamine- and cAMP-regulated phosphoprotein, DARPP-32. We also show that there is an inverse correlation between miR-142-3p levels and D1 receptor protein expression in the mouse brain during postnatal development. This is the first study to demonstrate that the post-transcriptional regulation of D1 receptor expression is mediated by microRNA-induced translational suppression.

## Introduction

The neurotransmitter dopamine binds and activates two major subfamilies of dopamine receptors. The D1-like subfamily includes D1 and D5 receptors and the D2-like subfamily includes D2, D3 and D4 receptors. The D1 dopamine receptor subtype is expressed at high levels in the basal ganglia and prefrontal cortex regions in humans and rodents [1]. In addition, several studies have shown that the D1 dopamine receptor is expressed in the kidneys [2] and lymphocytes [3]. D1 receptor signaling function has been extensively studied and it has been shown to activate adenylate cyclase and modulate ion channel function [4]. D1 receptors in the brain are involved in motor control and cognition and are essential for mediating addictive behaviors [1]. In the kidney, D1 receptors modulate the sodium-potassium ATPase and the sodium-hydrogen exchanger and regulate diuresis and natriuresis [5]. Recently, peripheral dopamine acting via D1 receptor expressed on antigen-presenting dendritic cells and T-cells was shown to modulate the differentiation of various types of T-cells following immune activation [3]. Thus the D1 receptor is expressed in the periphery and the brain and plays an important role in many physiological and pathophysiological conditions.

The expression of D1 receptor is plastic and its levels change during development, aging, pathological conditions and following chronic drug treatment [6], [7]. However, the molecular mechanisms and extracellular factors that regulate the expression of the D1 receptor gene are not well understood. We have previously shown that the expression of D1 receptor can be regulated at the transcriptional, post-transcriptional and post-translational level during neuronal differentiation [8]. We have also shown that extracellular factors such as brain derived neurotrophic factor (BDNF), NT-3 and adenosine regulates D1 receptor expression at the transcriptional level [8]–[10]. In the developing rat brain, the expression of D1 receptor mRNA begins to increase around embryonic day 14 and reaches steady state expression level around postnatal day 5; in contrast, D1 receptor protein levels increase postnatally reaching peak values between postnatal day 7 and 14 [6], [11]. This lack of correlation in expression of D1 receptor mRNA and protein is also seen during human brain development [12]. Interestingly, the expression of D1 receptor protein in lymphocytes also shows a similar age-dependent upregulation [13]. Together these studies suggest that D1 receptor expression is regulated at the post-transcriptional level during brain development and under pathological conditions. The molecular mechanisms that mediate the post-transcriptional regulation of D1 receptor expression are not known. In this paper, we tested the hypotheses that the expression of D1 receptor is post-transcriptionally regulated during early postnatal mouse brain development and that the D1 receptor post-transcriptional regulation is mediated via *trans*-acting factors binding *cis*-elements in the 3′-untranslated region (3′UTR) of the D1 receptor mRNA.

Using the mouse CATH.a-derived (CAD) catecholaminergic neuronal cell line, which endogenously expresses functional D1 dopamine receptor, we have previously shown multi-modal regulation of D1 receptor expression during serum withdrawal-induced neuronal differentiation [8]. In addition, the BDNF-mediated increase in endogenous D1 receptor expression in CAD cells also exhibits post-transcriptional regulation with the increase in D1 receptor protein being temporally delayed by greater than nine hours following the increase in D1 receptor mRNA levels [9]. Thus, the CAD cells are an ideal *in vitro* model to study the molecular mechanisms underlying post-transcriptional regulation of endogenously expressed D1 receptors. In this paper we demonstrate that the D1 receptor exhibits post-transcriptional regulation during postnatal mouse brain development and use the CAD cell line to identify the molecular mechanisms underlying D1 receptor post-transcriptional regulation. Using a systematic approach, we demonstrate that the D1 receptor 3′UTR is necessary and sufficient for D1 receptor post-transcriptional regulation. We demonstrate for the first time that the microRNA, miR-142-3p, directly regulates D1 receptor post-transcriptional regulation in CAD cells and that its expression is inversely correlated to D1 receptor protein expression during postnatal mouse brain development. Furthermore, specific inhibition of endogenous miR-142-3p in CAD cells increases D1 receptor protein levels and enhances D1 receptor mediated-signaling. This study is the first to report that a noncoding RNA-mediated translational suppression mechanism regulates the expression of D1dopamine receptors.

## Materials and Methods

### Animals and Brain Tissue Harvest

Male mice with a Swiss Webster/FVB genetic background were used in the study. The mice used in this study were obtained from locally bred animals kept on a 12∶12 hour, light-dark schedule (lights on at 0800) and provided ad libitum food and water. The animal protocols were approved by the IACUC committee at UMDNJ-New Jersey Medical School. Whole brain was isolated, sectioned on a Vibratome and the dorsal striatum, including the caudate-putamen region punched out from the appropriate slices. The punches for RNA isolation were stored in RNAlater® (Ambion) and those for protein analysis rapidly frozen in a dry ice-ethanol mixture and stored at −80°C.

### Cell Culture and Transfection

CAD cells were maintained in DMEM/F12 media, 8% fetal calf serum (FCS) and 100 U/mL penicillin/streptomycin. CAD cells used in the experiments were plated and grown in either 6- or 12-well tissue culture plates. CAD cells were plated and grown for 24 hours or more in serum-containing media to about 60% confluence before transfection. Differentiation and transfection of CAD cells were done as described previously [8]–[10]. For transfection, the Lipofectamine 2000 (Invitrogen) transfection reagent and test plasmid DNA’s were mixed in OPTI-MEM media and the mixture overlaid on non-differentiated CAD cells in serum media for six hours. After six hours, the media was replaced with fresh serum-containing or serum-free media and the cells harvested 48 hours later. The transfection efficiency was monitored by co-transfecting either a plasmid expressing the enhanced green fluorescent protein (EGFP) reporter gene or the Flag™_-_tagged bacterial alkaline phosphatase gene (BAP-Flag™). To inhibit microRNA function, anti-mirs that specifically targeted miR-142-3p (Ambion) were transfected at a concentration of 30 nM per well (for a 12-well plate). A FAM-labeled fluorescent anti-mir (Ambion) was used as a negative control. To knock-down Dicer levels in non-differentiated CAD cells, we transfected two different siRNAs that specifically targeted the mouse Dicer mRNA (Ambion) at a concentration of 10 nM per well (for a 12-well plate). A FAM-labeled fluorescent siRNA (Ambion) was used as a negative control. In functional experiments, endogenous miR-142-3p was also inhibited using a miRZip™ anti-sense microRNA expressing plasmid with anti-miR-142-3p activity (System Biosciences, Mountain View, CA, USA). For these functional experiments we also generated and used a negative control empty miRZip™ vector plasmid in which the nucleotides encoding the anti-sense miR-142-3p were deleted.

### Cloning, Deletion and Mutagenesis

The cloning of the 6400 bp mouse D1 receptor promoter has been previously described [9]. The mouse D1 receptor 3′UTR region (the 1277 bp and 1684 bp fragments) was amplified using specific primers and a BAC construct containing the entire mouse D1 receptor gene (BAC clone address RP23-47M2; Invitrogen). The primers included Not I and HindIII/AflII/PmlI restriction sites which facilitated the cloning of the amplified D1 3′UTR into the various reporter plasmids. The various deletion constructs were generated using PCR primer pairs that flanked the polyadenylation site within the 1277 bp D1 receptor 3′UTR. The primers used for generating the deletion constructs also contained the above restriction enzyme sites to facilitate cloning of the products into the reporter plasmids. The D1 receptor 3′UTR constructs with mutations in the microRNA binding sites were generated using a mutagenic primer with a KpnI restriction site that disrupted the individual microRNA seed recognition sequence (Figure S1). The three different microRNA binding site mutants were made in the context of a reporter construct that included the 1277 bp D1 receptor 3′UTR. In addition, wild-type and mutant D1 receptor 3′UTRs were individually subcloned into a reporter plasmid that included a heterologous bovine growth hormone 3′UTR. All recombinant plasmids were sequenced and the wild type D1 receptor 3′UTR sequence was found to match the sequence in the NCBI database. All plasmids were purified on two CsCl gradients prior to sequencing and transfection.

### Real-time Reverse Transcriptase PCR

RNA isolation and RT-PCR was performed as described previously [8]–[10]. Briefly RNA was isolated using Trizol® reagent (Invitrogen) or the RNeasy® Mini Kit (Qiagen) according to the manufacturers’ instructions. RT reaction was carried out using Superscript III RT. PCR was performed using the Roche Light Cycler (Indianapolis, IN, USA) in either the SYBR® Green format, using primers and conditions described previously, or using gene-specific TaqMan® gene expression assays (Applied Biosystems) [8]–[10]. The RT-PCR conditions were optimized as shown in Figure S2. Appropriate negative and positive controls were included in the RT-PCR experiments. For the SYBER® green method, the products were run on an agarose gel, stained with ethidium bromide. The captured images were inverted for visualization purposes.

### Western Blotting

Tissues and cells were harvested and lysed, solubilized using the Cellytic™-M or MT reagent (Sigma) supplemented with 1 mM phenylmethylsulfonylfluoride (PMSF) and 1% protease and phosphatase inhibitor cocktails (Sigma). Protein amounts in the lysates were determined using the BCA assay (BioRad, Hercules, CA, USA) and equal amounts of total cell proteins (10 to 100 µg) were loaded on to 10% gels and separated using SDS-PAGE. Blots were developed using appropriate primary and secondary antibodies as described previously [8]–[10]. Total and phosphorylated dopamine- and cAMP-regulated phosphoproteins (DARPP-32) were detected using rabbit monoclonal antibodies (Cell Signaling Technology, Danvers, MA, USA). For detecting D1 receptor protein, we used a rat monoclonal anti D1 receptor antibody (Sigma) as described previously [8]–[10]. This antibody specifically detects the various glycosylated forms of D1 receptor protein (Figure S2). Equal loading in lanes was determined by post-hoc staining of the nitrocellulose blots with the amido black total protein stain.

### β-galactosidase Assay

For the β-galactosidase assay, the CAD cell pellet was lysed for 10 minutes on ice in the β-gal lysis buffer (10 mM KCl, 1 mM MgSO_4_, 2.5 mM EDTA, 0.25% NP-40 detergent, 50 mM β-mercaptoethanol and 100 mM sodium phosphate buffer [pH7.2]). The lysate was spun at 14,000×g for 10 minutes and the protein amount in the supernatant measured using the Bradford protein assay (Pierce). The β-galactosidase reporter enzyme activity in the lysate was detected using chlorophenol red-β-galactoside (CPRG, Roche) as a substrate. The β-galactosidase enzyme cleaves the CPRG substrate to yield a colored product which has maximal absorbance at 575 nm and thus can be detected using a visible wavelength spectrophotometer. The β-galactosidase reporter enzyme activity was normalized to the total protein amount present in the lysate and further normalized for transfection efficiency.

### cAMP Measurements

Cyclic AMP (cAMP) levels were measured using the Cyclic AMP XP® Assay Kit (Cell Signaling Technology). Briefly, CAD cells (10^6^ cells) were plated and transfected with negative control or miRZip™ 142-3p constructs in 12-well plates (Costar, Corning, NY, USA) and cultured for 48 hrs. To minimize variations due to differences in cell plating density or growth rate, we used two wells for each treatment variable. CAD cells were treated for 10 minutes at 37°C with 0.3 mM IBMX (phosphodiestrase inhibitor), or 0.3 mM IBMX + increasing concentrations (0.03 µM to 10 µM) of D1 receptor agonist, A77636 (Sigma), and lysed in 12 well plates. Sample lysates from the two wells were pooled and 0.05 ml transferred into three independent wells of the 96-well EIA plate. The cAMP levels were measured by following the recommendations of the kit manufacturer (Cell Signaling Technology). The final absorbance values were measured at 450 nm on a Model 680 Microplate Reader (BioRad). The amount of cAMP in each well was determined by comparing the absorbance values of the treated variables with a series of standards provided with the kit. The cAMP level in each well was normalized to total protein present in each well. Protein levels were measured using the bicinchoninic acid (BCA) assay (Thermo Fisher Scientific, Rockford, Illinois, USA). For each experiment, the standards were assayed in parallel and used to generate a standard curve. The cAMP levels in each treated sample were assayed in triplicate.

### Statistics

Experiments were repeated at least three independent times with the specific number of repeats indicated in the individual figure legends. One-way Analysis of variance (ANOVA) and post-hoc Student-Newman-Keuls (SNK) tests and two-tailed Student’s t-test were performed with the SigmaPlot® 11 software (SPSS Inc.). Data were considered statistically significant when the probability value (P) was less than 0.05.

## Results

### D1 Dopamine Receptor is Post-transcriptionally Regulated during Postnatal Mouse Brain Development

**Figure 1:**
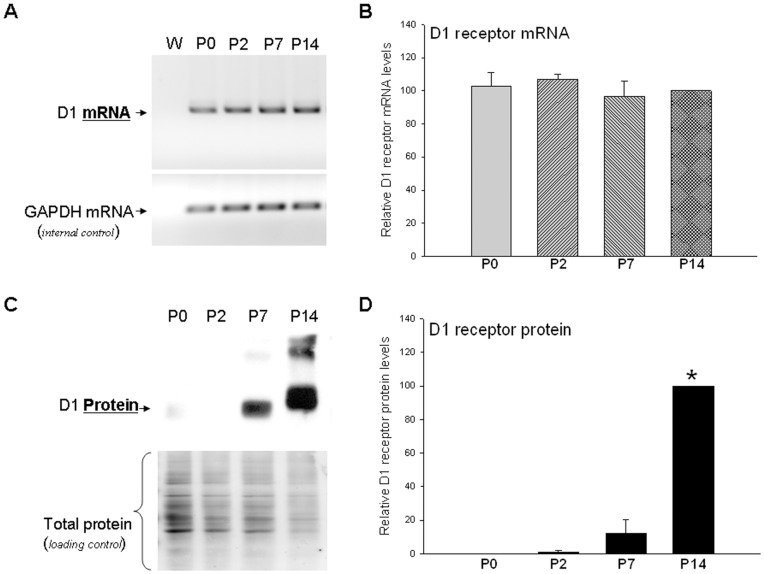
D1 dopamine receptor exhibits post-transcriptional regulation during early postnatal mouse brain development. (*A*) Representative RT-PCR gel showing D1 receptor and internal control GAPDH mRNA levels in mouse brain striatum isolated from new born animals (P0) through postnatal day 14 (P14). The real-time RT-PCR was performed using the SYBR® green method and the reactions stopped in the exponential phase. (*B*) Cumulative data showing D1 receptor mRNA levels normalized to GAPDH internal control mRNA levels in the striatal region isolated from mice that were P0 through P14. The data are from real-time RT-PCR experiments using TaqMan® probes specific for mouse D1 receptor and GAPDH. (*C*) Representative western blot showing D1 receptor protein levels in mouse brain striatum isolated from animals that were P0 through P14. The multiple bands correspond to different glycosylated forms of the D1 receptor [30], [31]. The lower panel shows amido black staining of the membrane in the upper panel and shows the total protein loaded in each lane. (*D*) Cumulative data showing D1 receptor protein levels normalized to total protein. The plotted bars represent the mean values ± s.e.m. (n = 5). *P<0.002, significant difference between P14 and all other ages, Kruskal-Wallis One Way ANOVA on Ranks, post hoc SNK test.

Previous studies in rats and humans have suggested that the D1 dopamine receptor exhibits post-transcriptional regulation during brain development [6], [11], [12]. Figure 1 shows the postnatal expression profile of D1 receptor mRNA and protein during postnatal brain development in mice. There is no significant difference in the expression of D1 receptor mRNA between day of birth (P0) through postnatal day 14 (P14) (Fig. 1A and 1C); in contrast, the relative expression of D1 receptor protein is low at P0 and begins to increase at P7 with a significant increase in expression on P14 (Fig. 1B and 1D). The specificity and sensitivity of the TaqMan® probe and monoclonal antibody used to detect D1 receptor expression is shown in Figure S1A–C. The D1 dopamine receptor is glycosylated and the multiple bands in the gel represents various glycosylated forms of the D1 receptor. Together the results in Figure 1 suggest that D1 receptor expression is post-transcriptionally regulated during mouse brain development with a significant upregulation of mature D1 receptor protein on P14.

### D1 Dopamine Receptor is Post-transcriptionally Regulated in the Mouse CAD Catecholaminergic Cell Line

**Figure 2:**
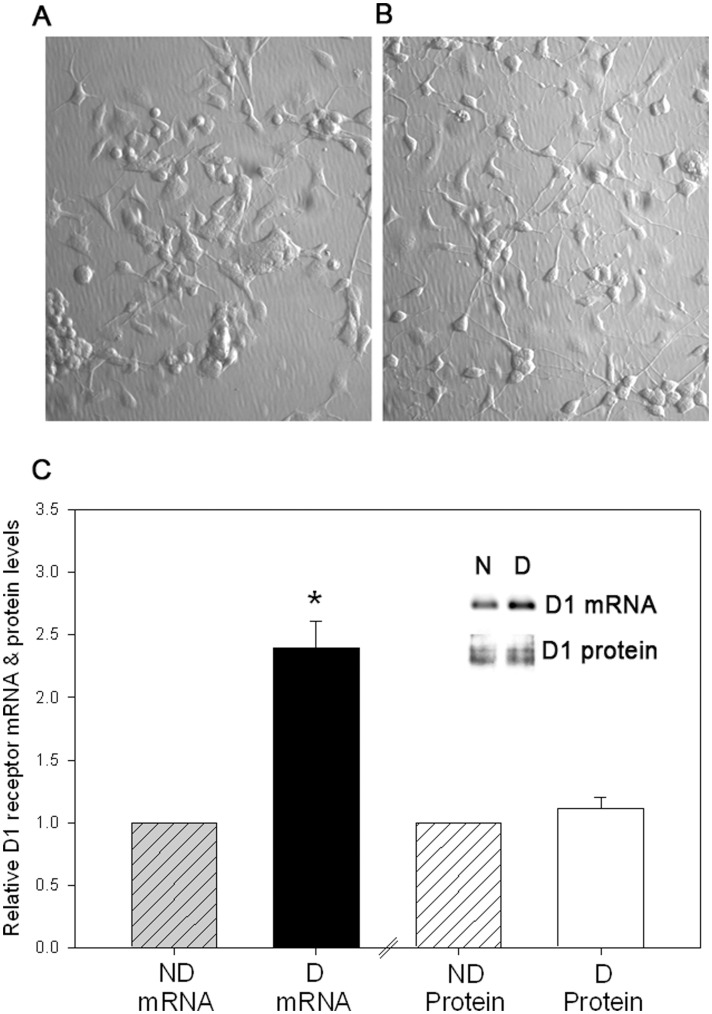
Endogenous D1 dopamine receptor exhibits post-transcriptional regulation in the mouse CAD catecholaminergic cell line. (*A*) Representative images of non-differentiated (ND) and (*B*) differentiated (D) CAD cells. (*C*) Cumulative data showing relative D1 receptor mRNA and protein in non-differentiated (ND) and differentiated (D) CAD cells. The mRNA levels were measured using gene-specific TaqMan® probes and the D1 receptor protein levels detected using the rat monoclonal antibody. The D1 receptor mRNA levels were normalized to GAPDH mRNA levels and D1 receptor protein was normalized to total protein. The inset shows a representative gel with D1 receptor mRNA and D1 receptor protein in non-differentiated (N) and differentiated (D) CAD cells. The plotted bars represent the mean values ± s.e.m. (n = 5). *P<0.001, significant difference in D1 receptor mRNA levels between non-differentiated and differentiated CAD cells, two-tailed pair-wise Student’s t-test.

The CAD cell line, derived from the CATH.a catecholaminergic cell line, is a mouse neuronal cell line that undergoes reversible morphological differentiation upon serum withdrawal (Fig. 2A and 2B) [14]. We have previously shown that the CAD cells express endogenous D1 receptor mRNA that yields functional D1 receptor protein [8]; however, compared to striatal tissue, the expression of D1 receptor protein is relatively low (Figure S2D). This suggests that D1 dopamine receptor is post-transcriptionally regulated in nondifferentiated CAD cells. Furthermore, consistent with our previous studies [8], the endogenous D1 dopamine receptor mRNA in CAD cells is significantly upregulated upon differentiation induced by serum withdrawal; however, the increase in D1 receptor mRNA is not accompanied by a concomitant increase in D1 receptor protein (Fig. 2C). The results suggest that the endogenous D1 receptor expression is post-transcriptionally regulated in non-differentiated and differentiated CAD cells, providing a convenient expression system to determine the molecular mechanisms underlying D1 receptor post-transcriptional regulation.

### The 3′untranslated Region of D1 Receptor mRNA is Necessary and Sufficient for Post-transcriptional Regulation of D1 Receptor Expression

**Figure 3:**
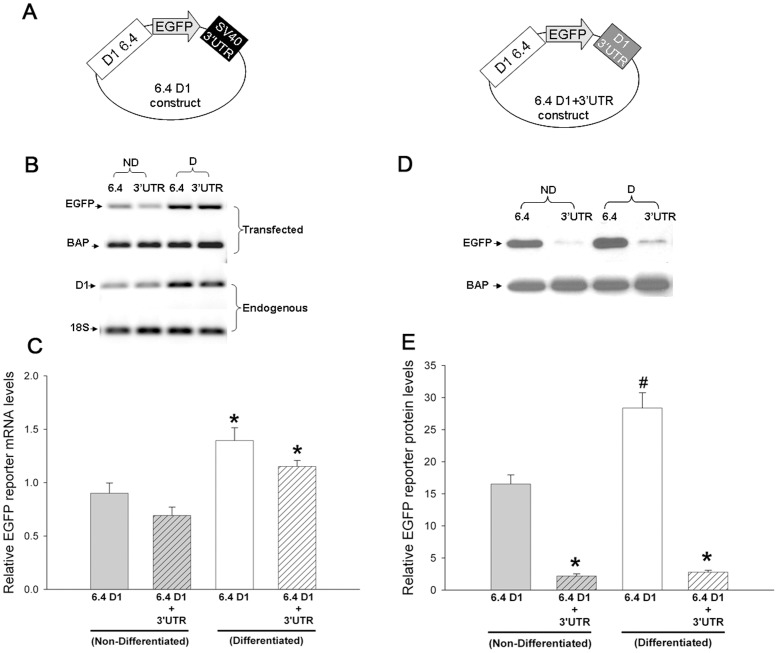
Reporter gene regulated by the mouse D1 receptor promoter and 3′UTR exhibits post-transcriptional regulation. (*A*) Schematic representation of the two EGFP-reporter constructs used. One construct (6.4 D1) has the 6.4 kb D1 receptor promoter and the heterologous SV40 3′UTR; the other construct (6.4 D1+3′UTR) has the 6.4 kb D1 receptor promoter and the 1277 bp D1 3′UTR. (*B*) Representative real-time RT-PCR gel using the SYBER® green format showing the mRNA levels of EGFP reporter, BAP-Flag™ (transfection control), endogenous D1 receptor and 18S ribosomal RNA (internal control) in non-differentiated (ND) and differentiated (D) CAD cells harvested 48 hours after transient transfection of the 6.4D1 or the 6.4 D1+3′UTR reporter constructs. The PCR reactions were stopped in the exponential phase and the products run out on the gel. (*C*) Cumulative data showing normalized EGFP reporter mRNA levels from ND and D CAD cells transfected with the 6.4 D1 or the 6.4 D1+3′UTR constructs. The EGFP mRNA levels were normalized to co-transfected BAP-Flag™ mRNA and the endogenous 18S ribosomal RNA. The bars represent the mean values ± s.e.m. (n = 7). *P<0.004, significant difference between ND and D CAD cells transfected with either reporter constructs, One Way ANOVA, post hoc SNK test. (*D*) Representative western blot gel showing the protein levels of EGFP reporter, BAP-Flag™ (transfection control) in ND and D CAD cells harvested 48 hours after transient transfection of the 6.4 D1 or 6.4 D1+3′UTR reporter constructs. (*E*) Cumulative data showing normalized EGFP reporter protein levels from ND and D CAD cells transfected with the 6.4 D1 or the 6.4 D1+3′UTR constructs. The EGFP protein levels were normalized to co-transfected BAP-Flag™ protein and total protein levels in the lysate. The bars represent the mean values ± s.e.m. (n = 7). *P<0.001, significant difference between CAD cells transfected with the 6.4 D1 or the 6.4 D1+3′UTR constructs in either ND or D CAD cells, ^#^, P<0.001, significant difference between ND and D CAD cells transfected with the 6.4 D1 reporter construct, One Way ANOVA, post hoc SNK test.

To determine the post-transcriptional regulatory mechanisms of D1 receptor expression, we cloned a 6.4 kb upstream D1 receptor promoter region that included the 5′ untranslated region (UTR) of the D1 receptor mRNA [9]. Separately, we also cloned the 1277 bp 3′UTR present in the D1 receptor mRNA. The 6.4 kb upstream D1 promoter region including the 5′UTR (6.4 D1) was fused to an EGFP reporter gene (Fig. 3A). In a second construct, we subcloned the 1277 bp 3′UTR of the D1 receptor downstream of the EGFP reporter gene, replacing the recombinant SV40 3′UTR present in the vector of the 6.4 D1 construct (6.4 D1+3′UTR; Fig. 3A). The 1277 bp 3′UTR of the D1 receptor gene contains a single polyadenylation signal (AAUAAA). The two constructs were separately transfected into CAD cells along with a bacterial alkaline phosphatase (BAP)-Flag™ construct to serve as an internal control for transfection efficiency. Twelve hours after transfection, half of the transfected cells were switched to the serum-free differentiation media to induce CAD cell differentiation. All cells were harvested 48 hours later and the level of EGFP reporter mRNA, BAP-Flag™ mRNA, endogenous D1 receptor mRNA and 18S ribosomal RNA levels were measured using real-time RT-PCR with Syber green®. The PCR reactions were stopped in the exponential phase and the products run out on a gel. In parallel, half of the harvested cells were used for protein analysis by western blot using antibodies specific for the EGFP protein and the Flag™ epitope. The results in Figure 3B and 3C show that differentiation of CAD cells increased expression levels of not only the endogenous D1 receptor mRNA, but also the EGFP reporter mRNA generated from the transfected 6.4 D1 and 6.4 D1+3′UTR constructs. These results suggest that the 6.4 kb D1 promoter region in the EGFP reporter construct contains the sequences required for the differentiation-induced increase in D1 receptor mRNA. In addition, the presence of the D1 receptor 3′UTR in the reporter construct did not affect its ability to increase mRNA expression following differentiation.

To evaluate the ability of the reporter construct to mimic the post-transcriptional regulation of the endogenous D1 receptor gene, we compared the expression of EGFP reporter mRNA to EGFP reporter protein in transfected CAD cells. The level of EGFP reporter mRNA generated by the 6.4 D1 and 6.4 D1+3′UTR constructs under either nondifferentiated or differentiated conditions were equal (Fig. 3B and 3C), suggesting that the introduction of the D1 receptor 3′UTR into the 6.4 D1 EGFP reporter construct had no effect on the level of steady-state EGFP reporter mRNA. In contrast to the results with mRNA, the levels of EGFP reporter protein from the 6.4 D1 and 6.4 D1+3′UTR constructs were significantly different. Figures 3D and 3E show that introduction of the D1 receptor 3′UTR significantly repressed the expression of EGFP reporter protein in both non-differentiated and differentiated CAD cells by greater than 15-fold. Together the results in Figure 3 suggest that sequences in the D1 receptor 3′UTR represses translation. Analysis of the nucleotide sequences downstream of the D1 receptor stop codon identified a second poly adenylation signal downstream of the first poly adenylation signal (Figure S3A). We compared the D1 receptor post-transcriptional regulation in reporter constructs containing D1 receptor 3′UTR that was either 1277 bp or 1684 bp long and found that both 3′UTR’s suppressed the translation of the EGFP reporter protein equally (Figure S3B). This suggested that the D1 receptor post-transcriptional regulation is mediated by sequences in the 1277 bp D1 receptor 3′UTR proximal to the stop codon.

**Figure 4:**
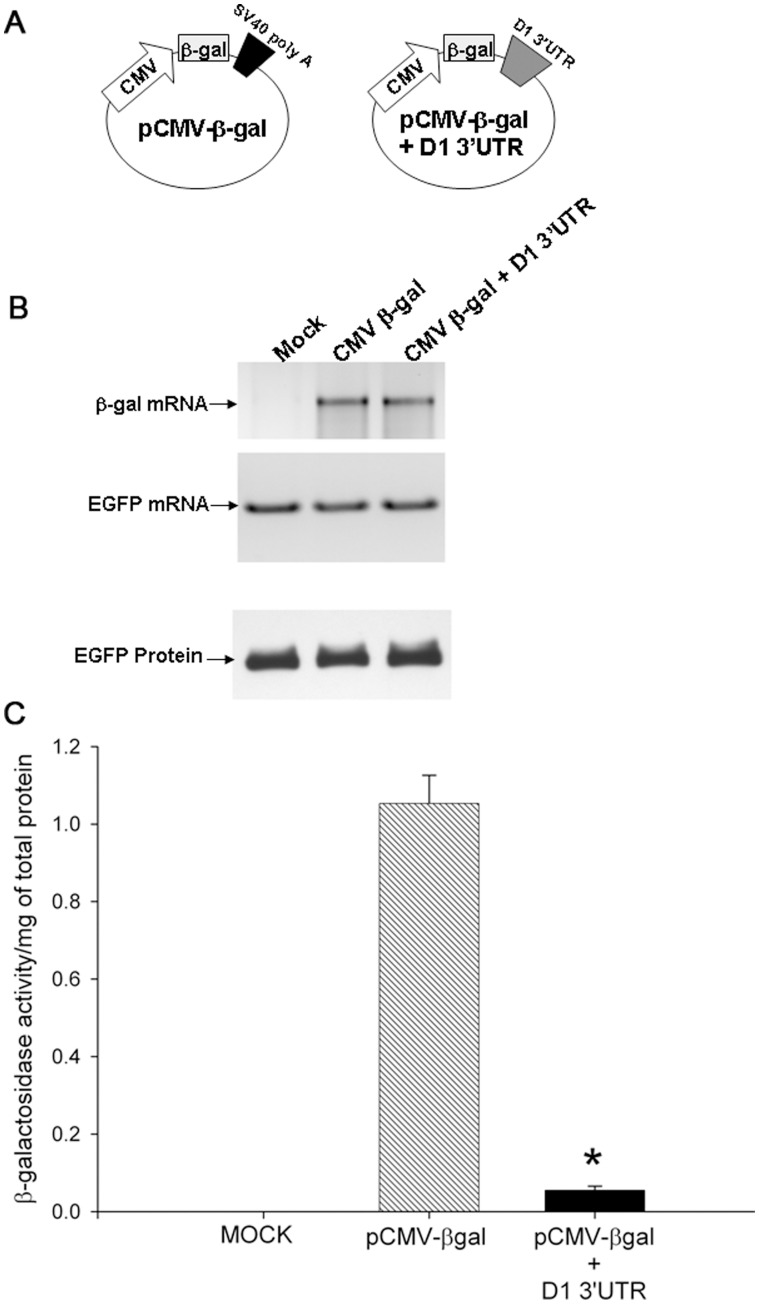
The D1 3′UTR is necessary and sufficient for D1 receptor post-transcriptional regulation. (*A*) Schematic representation of reporter plasmids with the β-galactosidase (β-gal) reporter gene regulated by the constitutively active cytomegalovirus (CMV) promoter. In one of the constructs, the recombinant 3′UTR with the SV40 polyadenylation (poly A) sequence was replaced by the 1277 bp D1 receptor 3′UTR. (*B*) Non differentiated CAD cells were transfected with either control pcDNA plasmid (Mock), the positive control pCMV β-gal plasmid with SV40 poly A (pCMV β-gal) or the plasmid containing CMV β-gal and the 1277 bp D1 receptor gene 3′UTR (pCMV β-gal+D1 3′UTR). The cells were also co-transfected with a plasmid expressing EGFP to control for transfection efficiency. The β-gal and EGFP mRNA levels (upper panels) were measured using real time RT-PCR and the products were run out on an agarose gel. Also shown is the EGFP protein level in the transfected cells. (*C*) The β-gal reporter enzyme activity was normalized to total protein amounts. The bars represent the mean values ± s.e.m. (n = 3). The enzyme activity was significantly reduced in cells transfected with CMV β-gal+D1 3′UTR compared to cells transfected with parental CMV β-gal plasmid (*, P<0.001, two-tailed pair-wise Student’s t-test).

We next determined if the D1 receptor 3′UTR was necessary and sufficient to repress the translation of a reporter mRNA that was generated from a heterologous promoter such as the cytomegalovirus (CMV) promoter. We introduced the 1277 bp D1 receptor 3′UTR region into the pCMV β-galactosidase reporter construct (Fig. 4A). The resultant pCMV β-gal+D1 3′UTR and the parental pCMV β-gal constructs were independently transfected into non-differentiated CAD cells along with a plasmid expressing the EGFP as a control for transfection efficiency. Forty eight hours after transfection, cells were harvested and β-galactosidase reporter mRNA and enzyme activity determined (Fig. 4B and 4C). The β-galactosidase reporter and EGFP mRNA levels were measured using real time RT-PCR. The β-galactosidase reporter enzyme activity was detected using CPRG as a substrate. The results in Figure 4 show that the β-galactosidase reporter mRNA levels in cells transfected with pCMV β-gal and pCMV β-gal+D1 3′UTR were identical. Yet the β-galactosidase activity (Fig. 4C) levels were reduced ∼ 20-fold in cells transfected with pCMV β-gal+D1 3′UTR construct. These results strongly suggest that, in CAD cells, the D1 receptor 3′UTR is necessary and sufficient to suppress the translation of mRNA generated from a heterologous promoter. Taken together, these data strongly suggest that the D1 receptor 3′UTR is necessary and sufficient for the post-transcriptional regulation of D1 receptor expression.

### MicroRNA 142-3p Directly Mediates the D1 Receptor Post-transcriptional Regulation in CAD Cells

**Figure 5:**
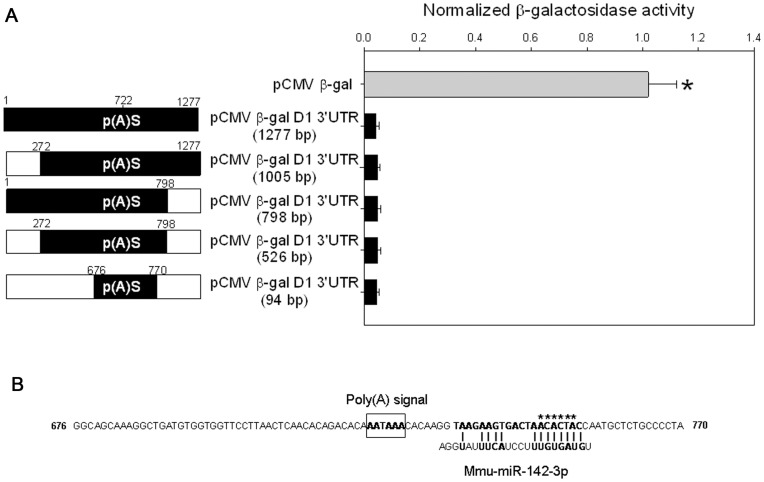
A 94 bp fragment of the D1 receptor 3′UTR is necessary and sufficient for post-transcriptional regulation. (*A*) Relative β-galactosidase activity, normalized to total protein, in non-differentiated CAD cells transfected with the parent pCMV β-gal plasmid, pCMV β-gal+D1 3′UTR or the various pCMV β-gal+D1 3′UTR constructs with deletions in the 3′UTR. The schematic representation of the 1277 bp D1 3′UTR is shown with the white regions representing the deleted segments. All deletion constructs retained the poly adenylation signal (p(A)S). The β-gal reporter enzyme activity was normalized to total protein amounts. The bars represent the mean values ± s.e.m. (n = 5). *P<0.028, the enzyme activity was significantly reduced in cells transfected with CMV β-gal+D1 3′UTR and the various deletions constructs when compared to cells transfected with parental CMV β-gal plasmid, Kruskal-Wallis One Way ANOVA on Ranks, post hoc SNK test. (*B*) The nucleotide sequence of the 94 bp D1 receptor 3′UTR that retained post-transcriptional regulation. The poly adenylation signal and the consensus binding site for microRNA miR-142-3p are shown. The * indicate the nucleotides in the seed recognition sequence that was mutated using site directed mutagenesis for the experiment in Figure 6C and 6D.

The D1 receptor post-transcriptional regulation could be mediated by decreased mRNA stability, decreased protein stability or translational repression. The above results showing equivalent steady state mRNA levels of the endogenous D1 receptor and the two reporter genes (EGFP and β-galactosidase), taken together with our previous study showing no difference in D1 receptor mRNA stability in nondifferentiated and differentiated CAD cells [8], suggested that D1 receptor post-transcriptional regulation was not mediated by decreased mRNA stability. Similarly the observation that D1 receptor 3′UTR post-transcriptionally regulates endogenous D1 receptor protein as well as two dissimilar reporter proteins (EGFP and β-galactosidase) suggested that decreased protein stability was unlikely to mediate D1 receptor post-transcriptional regulation. To identify the molecular mechanism mediating translational repression, we created deletion constructs using the pCMV β-gal+D1 3′UTR parent construct in which the proximal and distal sequences flanking the poly adenylation site in the D1 receptor 3′UTR were deleted. The constructs with the D1 receptor 3′UTR deletions were transfected into non-differentiated CAD cells and β-galactosidase mRNA and activity measured 48 hours post-transfection. The results in Figure 5A show that the smallest deletion construct (pCMV β-gal+D1 3′UTR [94 bp]) continued to express low levels of β-galactosidase activity, suggesting that the 94 bp D1 receptor 3′UTR was necessary and sufficient for post-transcriptional regulation. There was no significant difference in the β-galactosidase mRNA levels expressed from these deletion constructs (Figure S4A and S5). Sequence analysis of the 94 bp D1 receptor 3′UTR fragment revealed a consensus binding site for the highly conserved microRNA, miR-142-3p (Fig. 5B).

**Figure 6:**
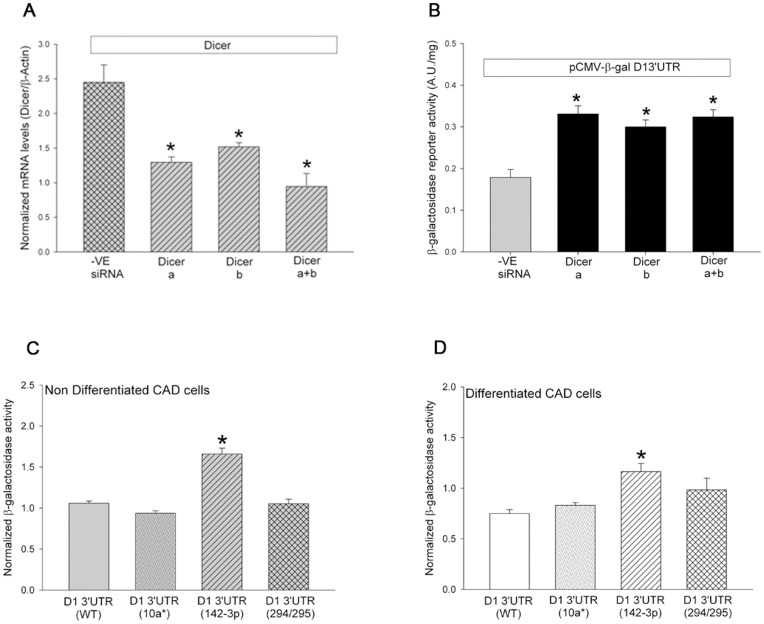
D1 receptor post-transcriptional regulation is mediated by microRNA miR-142-3p. (*A*) Levels of Dicer mRNA (normalized to β-actin) in CAD cells transiently-transfected with the pCMV- β-gal+D1 3′UTR construct and either the negative control siRNA (-ve siRNA) or two different siRNAs (Dicer “a” and Dicer “b”) targeting the dicer mRNA. The Dicer siRNAs were transfected individually or together (a+b) into non-differentiated CAD cells. The final concentration of the siRNA in the transfection was 10 nM. The cells were harvested 48 hours post transfection and mRNA levels measured using real-time RT-PCR with TaqMan™ probes for dicer and β-actin. The bars represent the mean values ± s.e.m. (n = 3 to 6). *P<0.001, the level of dicer mRNA was significantly reduced in cells transfected with the dicer siRNAs when compared to cells transfected with negative control siRNA, One Way ANOVA, post hoc SNK test. (*B*) Part of the harvested cells from the experiments in panel *A*, was lysed and the β-galactosidase reporter activity measured and normalized to total protein. The bars represent the mean values ± s.e.m. (n = 3 to 6). *P<0.001, the level of β-galactosidase reporter activity was significantly increased in cells transfected with the dicer siRNAs when compared to cells transfected with negative control siRNA, One Way ANOVA, post hoc SNK test. Wild type pCMV- β-gal+D1 3′UTR construct or pCMV- β-gal+D1 3′UTR constructs with mutations in the binding site for miR-10a*, miR-142-3p or miR-294/295 were transfected into non-differentiated (*C*) or differentiated (*D*) CAD cells and 48 hours later β-galactosidase reporter activity was measured and normalized to total protein. The bars represent the mean values ± s.e.m. (n = 6). *P<0.001, the level of β-galactosidase reporter activity was significantly increased in cells transfected with the pCMV- β-gal+D1 3′UTR construct with the miR-142-3p binding site mutated when compared to other transfected cells, One Way ANOVA, post hoc SNK test.

To determine if the translational repression of D1 receptor was mediated by microRNA, we first used siRNAs to reduce the expression of Dicer, the protein involved in the processing of microRNA precursors. The results in Figure 6A and 6B show that the transient knockdown of Dicer expression in nondifferentiated CAD cells significantly relieved the translational repression of the β-galactosidase reporter activity expressed from the pCMV β-gal+D1 3′UTR construct. Control experiments showed that the siRNA-mediated knockdown of Dicer expression had no effect on β-galactosidase expression from the parent pCMV β-gal construct (data not shown). These results suggested that D1 receptor post-transcriptional regulation might be mediated by Dicer-processed microRNAs.

**Figure 7:**
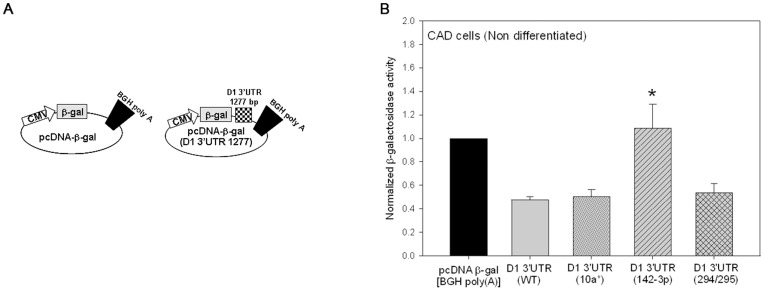
D1 receptor post-transcriptional regulation is mediated by D1 receptor 3′UTR and miR-142-3p even in the presence of a heterologous 3′UTR polyadenylation signal. (*A*) Schematic representation of the parent pcDNA- β-gal construct with the bovine growth hormone (BGH) 3′UTR and polyadenylation signal. Also shown is the recombinant pcDNA- β-gal (D1 3′UTR 1277) construct in which the 1277 bp D1 receptor 3′UTR was introduced between the stop codon of the β-galactosidase gene and the heterologous BGH 3′UTR. (*B*) The parent pcDNA- β-gal construct, the wild type pcDNA- β-gal (D1 3′UTR 1277) construct or pcDNA- β-gal (D1 3′UTR 1277) constructs with mutations in the binding site for miR-10a*, miR-142-3p or miR-294/295 were transiently-transfected into non-differentiated CAD cells and 48 hours later β-galactosidase reporter activity was measured and normalized to total protein. The bars represent the mean values ± s.e.m. (n = 6). *P<0.001, the level of β-galactosidase reporter activity was significantly increased in cells transfected with the pcDNA- β-gal (D1 3′UTR 1277) construct with the miR-142-3p binding site mutated when compared to cells transfected with wild type pcDNA- β-gal (D1 3′UTR 1277) or the other two mutant constructs, One Way ANOVA, post hoc SNK test.

Given the results of the deletion experiments in Figure 5A and the Dicer knock-down experiments in Figure 6, we generated pCMV β-gal+D1 3′UTR (1277 bp) constructs in which we specifically mutated nucleotides in the 1277 bp D1 receptor 3′UTR that were complementary to the seed sequence of microRNA miR-142-3p (Fig. 5B) and two other microRNAs (miR-294/295 and 10a*). The latter microRNAs have conserved consensus binding sites in the D1 receptor 3′UTR and served as negative controls. Rather than deleting the entire miRNA binding site, we took a conservative approach, and mutated the seed sequence binding sites for the three microRNAs (Figure S1) in the D1 receptor 3′UTR. The individual mutated pCMV β-gal+D1 3′UTR constructs were transfected into non-differentiated and differentiated CAD cells and β-galactosidase mRNA and activity measured 48 hours post-transfection. The results show that mutation of the miR-142-3p recognition site in the 1277 bp D1 receptor 3′UTR, but not the 10a* or 294/295 sites, significantly increased the β-galactosidase reporter enzyme activity in both non-differentiated and differentiated CAD cells. Control experiments showed that mutation of these binding sites had no significant effect on the β-galactosidase mRNA levels from the mutated pCMV β-gal+D1 3′UTR constructs (Figure S4B). To further confirm these results and to determine if the miR-142-3p translation repression was maintained in the context of a heterologous 3′UTR, we cloned the 1277 bp wild type and the three mutated D1 receptor 3′UT regions into a β-galactosidase expression plasmid with a heterologous bovine growth hormone (BGH) poly adenylation site (Fig. 7A). The resultant constructs were transfected into non-differentiated CAD cells and the β-galactosidase activity measured 48 hours post-transfection. The results confirmed that the D1 receptor 3′UTR repressed the translation of the β-galactosidase reporter protein in the presence of a heterologous poly adenylation site and this repression was abolished by specifically mutating the recognition site for miR-142-3p (Figure 7B).

**Figure 8:**
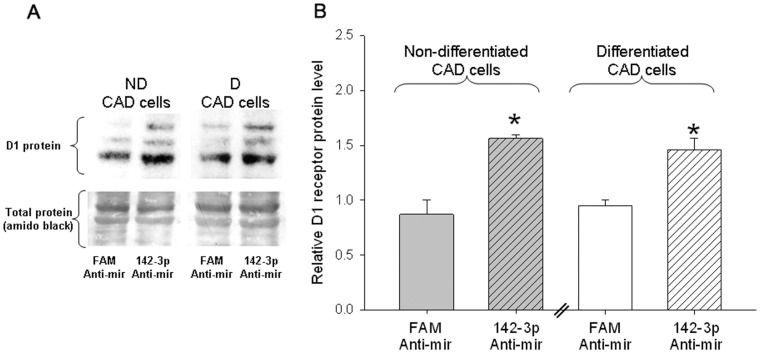
Inhibition of miR-142-3p increases endogenous D1 receptor protein expression in CAD cells. Representative western blot (*A*) and cumulative data (*B*) showing the levels of D1 receptor protein normalized to total protein in non-differentiated (ND) and differentiated (D) CAD cells 48 hours post-transient transfection with 30 nM per well (in 12-well plate) FAM-labeled negative control anti mir (FAM Anti-mir) or mmu-miR-142-3p anti-mir. The D1 receptor undergoes glycosylation and the three bands in the gel different glycosylated forms of the receptor. The bars represent the mean values ± s.e.m. (n = 3). The endogenous D1 receptor protein level was significantly increased in non-differentiated (*, P<0.006) and differentiated (*, P<0.011) CAD cells transfected with miR-142-3p anti-mir compared to cells transfected with control FAM Anti-mir (two-tailed pair-wise Student’s t-test).

To determine if miR-142-3p modulates the expression of endogenous D1 receptor protein in CAD cells, we transiently-transfected anti-mirs that specifically targeted miR-142-3p and measured endogenous D1 receptor protein levels. The results in Figure 8 show that anti-mir mediated inhibition of miR-142-3p significantly increased D1 receptor protein levels in non-differentiated and differentiated CAD cells when compared to a FAM-labeled negative control anti-mir. Taken together, these results strongly suggest miR-142-3p directly regulates the D1 receptor 3′UTR-mediated translational repression of D1 receptor expression in CAD cells.

### Antisense-mediated Inhibition of miR-142-3p in CAD Cells Enhances D1 Receptor Signaling Function

**Figure 9:**
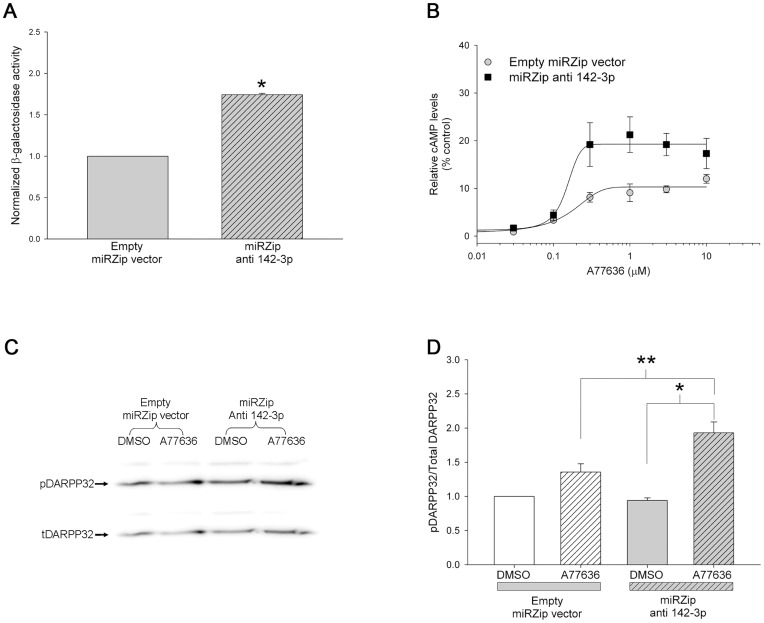
Inhibition of miR-142-3p enhances D1 receptor signaling in CAD cells. (*A*) Relative β-galactosidase activity, normalized to total protein, in non-differentiated CAD cells co-transfected with pCMV β-gal+D1 3′UTR (1277 bp) and either the control empty miRZip™ plasmid (gray bar) or the miRZip™ anti-142-3p plasmid (gray hatched bar). The cells were harvested 48 hours post-transfection. The bars represent the mean values ± s.e.m. (n = 3). *P<0.05, the enzyme activity was significantly increased in cells transfected with miRZip™ anti-142-3p plasmid when compared to cells transfected with control empty miRZip™ plasmid (two-tailed pair-wise Student’s t-test). (*B*) Relative cAMP levels in non-differentiated CAD cells transfected with the control empty miRZip™ plasmid (gray circles) or the miRZip™ anti-142-3p plasmid (black squares) that were treated with increasing concentration of D1 receptor agonist, A77636, and 0.3 mM IBMX for 10 minutes. The cells were treated 48 hours post-transfection. The levels of cAMP were normalized to total protein and plotted relative to control cells that were only treated with 0.3 mM IBMX. The bars represent the mean values ± s.e.m. (n = 3). Representative western blot (*C*) and cumulative data (*D*) showing the levels of phosphorylated and total DARPP-32 protein in non-differentiated CAD cells transfected with the control empty miRZip™ plasmid (white and white hatched bars) or the miRZip™ anti-142-3p plasmid (gray and gray hatched bars) that were treated with 10 µM A77636 and 0.3 mM IBMX for 30 minutes. The cells were treated 48 hours post-transfection. The bars represent the mean values ± s.e.m. (n = 4). *, **P<0.05, One Way ANOVA, post hoc SNK test.

The experiments in Figures 6, 7 and 8 suggested that mutating the miR-142-3p or knocking down miR-142-3p levels using anti-mirs increases the protein expression ∼80%. To determine if this increase was biologically significant, we next determined the effect of inhibiting endogenous miR-142-3p on endogenous D1 receptor signaling function in CAD cells. To decrease the levels of miR-142-3p, in these experiments we used the miRZip™ anti-sense 142-3p plasmid construct. Figure 9A shows that co-transfection of miRZip™ anti 142-3p plasmid with the pCMV β-gal+D1 3′UTR (1277 bp) into non-differentiated CAD cells significantly increased β-galactosidase reporter activity compared to cells co-transfected with empty miRZip™ vector plasmid and pCMV β-gal+D1 3′UTR (1277 bp). This result is consistent with above studies and suggested that the miRZip™ anti 142-3p plasmid also inhibits endogenous miR-142-3p and relieves D1 receptor post-transcriptional regulation in non-differentiated CAD cells.

Using the D1 receptor selective agonist, A77636, we have previously shown that D1 receptors in CAD cells couple to adenylate cyclases and increase intracellular cAMP levels upon agonist stimulation [8]–[10]. To determine the effect of inhibiting miR-142-3p on D1 receptor signaling function, we transfected non-differentiated CAD cells with miRZip™ anti 142-3p or empty miRZip™ vector plasmids and compared D1 receptor stimulated increase in intracellular cAMP levels. The results in Figure 9B shows that non-differentiated CAD cells transfected with miRZip™ anti 142-3p had significantly higher D1 receptor-agonist induced cAMP levels compared to cells transfected with the empty miRZip™ vector (∼85% increase in E_max_). The EC_50_ values for D1 receptor agonist, A77636, were similar (0.136 nM versus 0.142 nM for control and miRZip™ anti 142-3p, respectively) suggesting that the inhibition of miR-142-3p specifically affected E_max_. To further assess the biological significance of miR-142-3p regulation of D1 receptor expression, we evaluated the ability of D1 receptors to activate DARPP-32 in CAD cells transfected with miRZip™ anti 142-3p or empty miRZip™ vector plasmids. The results in Figures 9C and 9D shows that the levels of D1 receptor-activated phospho DARPP-32 is significantly higher in non-differentiated CAD cells transfected with miRZip™ anti 142-3p (∼102% increase) compared to empty miRZip™ vector plasmid (∼29% increase). Together these results strongly suggest that the miR-142-3p mediated regulation of D1 receptor protein expression is biologically significant.

### Micro RNA 142-3p Levels are Inversely Correlated to D1 Receptor Expression during Mouse Brain Development

**Figure 10:**
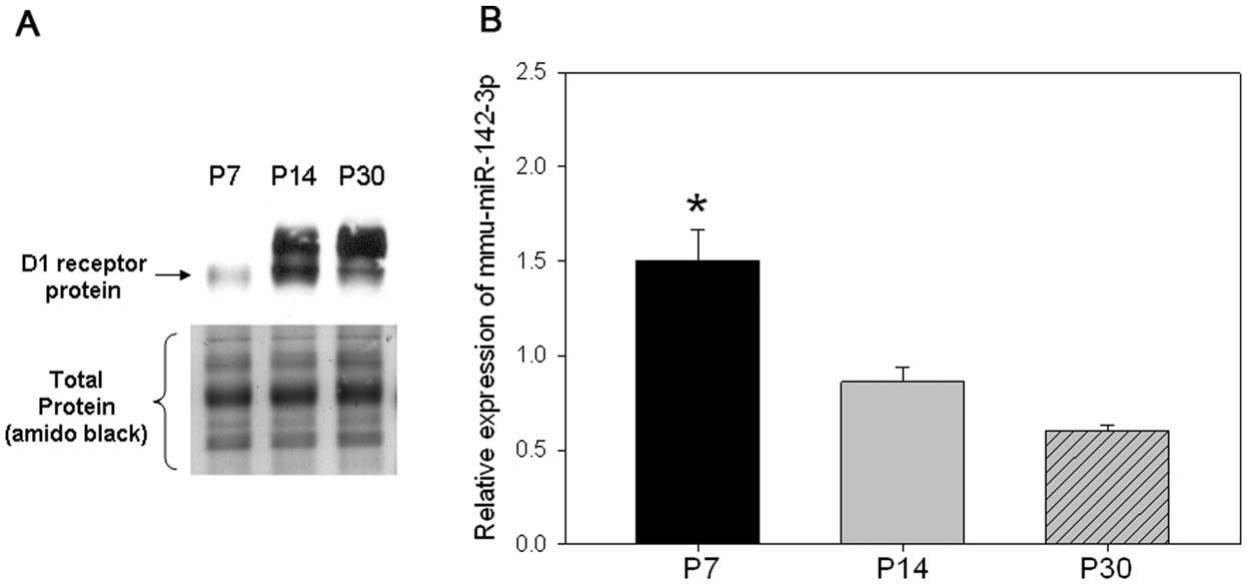
Expression level of miR-142-3p in postnatal striatal development is inversely correlated to D1 receptor protein expression. (*A*) Representative western blot which shows the level of D1 receptor protein in mouse brain striatum at P7, P14 and P30. Equal levels of total protein were loaded in each lane of the gel as evidenced by the equivalent amido black staining in the bottom panel. (*B*) Relative level of miR-142-3p normalized to U6 small nuclear RNA in striatal tissue punches at P7, P14 and P30. The bars represent the mean values ± s.e.m. (n = 3 to 4). *P<0.001, the level of miR-142-3p was significantly higher at P7 when compared to P14 and P30, One Way ANOVA, post hoc SNK test.

The results in Figure 1B and 1D showed that there is a significant increase in D1 receptor protein expression *in vivo* between P7 and P14 during mouse brain development. Figure 10A shows the increasing expression of D1 receptor protein at P7, P14 and P30 in the striatum region of mouse brain. We measured the expression of microRNA 142-3p in striatal brain tissue punches from P7, P14 and P30 using real-time RT-PCR. The result in Figure 10B shows that there is a significant reduction in miR-142-3p expression on P14 and P30 compared to P7 which inversely correlates with the increase in D1 receptor protein expression. These results suggest that miR-142-3p might be involved in the D1 receptor post-transcriptional regulation during postnatal mouse brain development.

## Discussion

The neurotransmitter dopamine controls many different physiological and behavioral functions in mammals through receptors that are members of seven transmembrane-spanning G-protein coupled receptor family. The D1 dopamine receptor is one of the major subtypes of dopamine receptors and is widely expressed in the brain and periphery. While numerous studies have extensively characterized D1 receptor function, mechanisms and factors that regulate its expression are less well characterized. The results presented in this paper establishes post-transcriptional regulation as a significant mechanism that controls the expression of D1 dopamine receptor protein during postnatal mouse brain development (Fig. 1) and in the mouse CAD catecholaminergic cell line (Fig. 2). We demonstrated that the D1 receptor 3′UTR was necessary and sufficient of mediating the post-transcriptional regulation (Figs. 3 and 4). The D1 receptor 3′UTR has not been defined; however comparison of the mouse D1 receptor 3′UTR, used in this study, to the rat and human D1 receptor 3′UTR revealed two poly adenylation signal sites 714 and 1635 nucleotides downstream from the stop codon. Our results suggest that the 1277 nucleotides long D1 receptor 3′UTR containing the first poly adenylation signal is necessary and sufficient for post-transcriptional regulation. Deletion and site-directed mutagenesis studies showed that the D1 receptor post-transcriptional regulation in CAD cells is mediated by a specific interaction of miRNA miR-142-3p with a single *cis*-element adjacent to the first poly adenylation signal site in the D1 receptor 3′UTR (Figs. 5, 6, and 7). Posttranscriptional regulation of many genes is mediated by miRNA. These ∼22 nucleotide long endogenous RNA molecules are partially complementary to nucleotide sequences in the 3′UTR of genes. By binding to the 3′UTR with partial complementarity, miRNA molecules have been reported to repress translation of the mRNA or affect mRNA stability. Previous studies have shown that miRNAs modulate expression of various G-protein coupled receptors including the β-adrenergic receptor [15], adenosine A2A receptor [16], serotonin receptors [17] and estrogen receptor α [18]. Our results add D1 dopamine receptors to the growing list of G-protein coupled receptors whose expression is regulated by miRNAs.

D1 receptors couple to activate adenylate cyclase, increasing intracellular cAMP levels. The increased cAMP activates the cAMP-dependent kinase which in turn phosphorylates the protein phosphatase DARPP-32. Activation of D1 receptors is known to increase phosphorylated DARPP-32 levels both *in vitro* and *in vivo*. The regulation of endogenous D1 receptor expression by miR-142-3p is biologically significant as inhibition of miR-142-3p increased endogenous D1 receptor protein levels (Fig. 8) and enhanced D1 receptor signaling as evidenced by increased cAMP production and phospo-DARPP-32 levels (Fig. 9). Finally, we showed that the expression of miR-142-3p was inversely correlated to D1 receptor protein expression during postnatal mouse brain development (Fig. 10). To our knowledge this is the first report of a microRNA mediated translational suppression of *any* of the five dopamine receptor subtypes. One previous study showed that miR-504 specifically targets a polymorphic site in human D1 3′UTR that is associated with nicotine dependence; however in that study miR-504 was found to increase, rather than decrease, the expression level of the human D1 receptor mRNA [19].

The identification of miR-142-3p as a regulator of D1 receptor expression was unexpected as the function of this microRNA has been previously described only in hematopoietic cells and lymphocytes were it was shown to modulate the differentiation of various classes of T-cells [20]. The expression of miR-142-3p is particularly high in the antigen-presenting dendritic cells [20]. Interestingly, lipopolysaccharide (LPS) treatment decreases the expression of miR-142-3p in dendritic cells, specifically relieving the translational repression of the interleukin-6 (IL-6) mRNA and increasing the level of secreted IL-6 [20]. Circulating levels of IL-6 is high in schizophrenic patients [21] and administration of IL-6 has been reported to increase stereotypic behavior in rodent models [22]–[24]. IL-6 along with TGF-β has also been shown to promote the differentiation of naive T-cells into the T-helper, Th-17 cells [3], [25], [26]. Interestingly, antagonizing D1 receptor function inhibits the differentiation of Th-17 cells [25]. Recent studies have shown that functional D1 dopamine receptors are expressed in dendritic cells and naive T-cells and the specific activation of these D1 receptors by dopamine promotes the differentiation of the T-cells into Th-2 and Th-17 cells [25]–[27]. An earlier study showed that D1 dopamine receptors suppress the differentiation of naïve T-cells into regulatory T-cells (T-regs) cells that are involved in the immune suppression response [28]. Our observations suggest that miR-142-3p mediated regulation of D1 receptor expression might contribute to T-cell differentiation.

While miR-142-3p function has been primarily described in the hematopoietic and immune systems, our results suggest that this microRNA is also expressed in the brain and in the neuronal CAD cell line. These results are consistent with a previous study which showed that mir-142-3p is not only expressed in the brain but is enriched in purified striatal post-synaptic densities along with the Argonaute protein-2, which is a key protein involved in microRNA-mediated post-transcriptional regulation [29]. This suggests that miR-142-3p-mediated post-transcriptional regulation might regulate translation of D1 receptor protein in dendritic spines. This novel molecular mechanism might dynamically regulate D1 receptor expression in the dendritic spines in response to neuronal excitation. Our novel finding that miR-142-3p is expressed in the brain and directly regulates the expression of D1 dopamine receptor protein suggests that miR-142-3p might play an important role in modulating D1 receptor-dependent behaviors.

## Supporting Information

Figure S1 Schematic representation of the binding sites for miR-294/295-3p, miR-142-3p and miR-10a* in D1 receptor 3′UTR and the seed recognition nucleotides that were targeted for mutation. Partial nucleotide sequence (from nucleotides 560 to 1250) of the 1277 bp D1 3′UTR that shows the putative binding sites (highlighted in yellow) for miR-294/295-3p, miR-142-3p and miR-10a*. The poly A signal site is indicated in bold letters. The top strand represents the wild type sequence and the bottom strand is the mutated version. The specific nucleotides that were mutated within each seed recognition sequence (underlined) are represented by a dash. Conserved nucleotides are represented by a *.
(TIF)

Figure S2 **Sensitivity and selectivity of molecular probes used to detect D1 dopamine receptor in this study.** (*A*) Raw traces from a representative real-time PCR detected using D1 receptor-specific TaqMan® probe shows the sensitivity of the amplification method to identify D1 receptor expression in serially diluted total mouse brain cDNA samples. (*B*) Cumulative data showing a linear correlation between the Ct values (cycle numbers) and concentration of serially diluted total mouse brain cDNA that were amplified and detected using TaqMan® probes specific for D1 dopamine receptor. The data points represent mean (± SEM) of 4 independent RT-PCR reactions and were fitted with linear regression. (*C*) Representative western blot showing the level of mouse D1 receptor protein in AtT-20 neuroendocrine cells (which does not express endogenous D1 receptor) transiently-transfected with empty plasmid vector (lane 1) or a constitutive expression plasmid encoding the mouse D1 dopamine receptor (lane 2). Each lane was loaded with 30 µg of total cell protein and the D1 receptor protein detected using the anti-D1 receptor rat monoclonal antibody. Multiple glycosylated forms of mature D1 receptor are detected with this antibody. (*D*) Representative western blot showing relative expression level of endogenous D1 receptor protein in non-differentiated CAD cells and mouse striatum. We loaded 100 µg of total protein from CAD cells in lane 1 and 1, 5, 10 and 30 µg of serially diluted total protein from the striatum of a mouse that was one month old in lanes 2 through 5. Two different film exposures as well as the amido black stained blot indicating total protein loaded in each lane is shown.
(TIF)

Figure S3 **D1 receptor post transcriptional regulation is mediated by the 1277 bp region proximal to the stop codon.** (*A*) Schematic representation of the two different D1 receptor 3′UTRs (1277 bp and 1684 bp) with the relative location of the two polyadenylation signals at positions 714 and 1635. (*B*) The two different D1 3′UTR regions were sub cloned into a construct with the EGFP reporter gene regulated by the 6400 bp D1 receptor promoter. The D1 receptor 3′UT regions replaced the heterologous SV40 3′UTR in the recombinant constructs. The three constructs were separately transfected into non-differentiated CAD cells along with a transfection control construct encoding the BAP-Flag™ gene. The inset shows equal EGFP reporter mRNA from all three constructs using real time RT-PCR. In contrast, western blot analysis with EGFP monoclonal antibody shows that EGFP reporter protein was significantly reduced in cells transfected with both D1 receptor 3′UTR constructs. The bars represent the mean values ± s.e.m. (n = 3). *P<0.001, One Way ANOVA, post hoc SNK test.
(TIF)

Figure S4 **The steady-state levels of β-galactosidase reporter mRNA from the D1 receptor 3′UTR constructs.** Constructs with deletions (*A*) or mutations in the microRNA binding sites (*B*) is not significantly different. The various reporter constructs were transiently transfected into non-differentiated CAD cells along with a control BAP-Flag™ construct for monitoring transfection efficiency. The mRNA levels were assayed using real-time RT-PCR with the SYBR® green method and primers specific for β-galactosidase and BAP-Flag™. The reactions were stopped in the exponential phase and the products run out on an ethidium bromide stained agarose gel.
(TIF)

Figure S5 **The steady-state levels of β-galactosidase reporter mRNA from the D1 receptor 3′UTR deletion constructs.** Cumulative results comparing β-galactosidase mRNA levels between non-differentiated CAD cells transfected with full length D1 3′UTR [pCMV β-gal+D1 3′UTR (1277 bp)] or D1 3′UTR constructs with deletions [pCMV β-gal+D1 3′UTR (526 bp) and pCMV β-gal+D1 3′UTR (94 bp)]. Constructs with deletions were not significantly different. The various reporter constructs were transiently transfected into non-differentiated CAD cells along with a control BAP-Flag™ construct for monitoring transfection efficiency. The mRNA levels were assayed using real-time RT-PCR with the SYBR® green method and primers specific for β-galactosidase and BAP-Flag™. The reactions were stopped in the exponential phase and the products run out on an ethidium bromide stained agarose gel (representative gel is shown in the inset). The bars represent the mean values ± s.e.m. (n = 3). Not significant, P>0.05, One Way ANOVA.
(TIF)

## References

[1] Missale C, Nash SR, Robinson SW, Jaber M, Caron MG (1998) Dopamine receptors: from structure to function. Physiol Rev 78(1): 189–225. 9457173 10.1152/physrev.1998.78.1.189

[2] Ozono R, O'Connell DP, Wang ZQ, Moore AF, Sanada H, et al (1997) Localization of the dopamine D1 receptor protein in the human heart and kidney. Hypertension 30: 725–729. 9323013 10.1161/01.hyp.30.3.725

[3] Pacheco R, Prado CE, Barrientos MJ, Bernales S (2009) Role of dopamine in the physiology of T-cells and dendritic cells. J Neuroimmunol 216(1–2): 8–19. 19732962 10.1016/j.jneuroim.2009.07.018

[4] Undieh AS (2010) Pharmacology of signaling induced by D1-like receptor activation. Pharmacol Therapeut 128: 37–60. 10.1016/j.pharmthera.2010.05.003 PMC2939266 20547182

[5] Hussain T, Lokhandwala MF (2003) Renal dopamine and hypertension. Exp Bio Med 228(2): 134–142. 10.1177/153537020322800202 12563019

[6] Jung AB, Bennett JP (1996) Development of striatal dopaminergic function. I. Pre- and postnatal development of mRNAs and binding sites for striatal D1 (D1a) and D2 (D2a) receptors. Brain Res Dev Brain Res 94(2): 109–120. 8836569 10.1016/0165-3806(96)00033-8

[7] Hurley MJ, Mash DC, Jenner P (2001) Dopamine D(1) receptor expression in human basal ganglia and changes in Parkinson's disease. Brain Res Mol Brain Res 87(2): 271–279. 11245931 10.1016/s0169-328x(01)00022-5

[8] Pasuit JB, Li Z, Kuzhikandathil EV (2004) Multi-modal regulation of endogenous D1 dopamine receptor expression and function in the CAD catecholaminergic cell line. J Neurochem 89(6): 1508–1519. 15189354 10.1111/j.1471-4159.2004.02450.x

[9] Do T, Kerr B, Kuzhikandathil EV (2007) Brain derived neurotrophic factor regulates the expression of D1 dopamine receptors. J Neurochem 100(2): 416–428. 17116228 10.1111/j.1471-4159.2006.04249.x

[10] Do T, Sun Q, Beuve A, Kuzhikandathil EV (2007) Extracellular cAMP inhibits D1 dopamine receptor expression in CAD catecholaminergic cells via A2a adenosine receptors. J Neurochem 101(3): 619–631. 17254022 10.1111/j.1471-4159.2006.04388.x

[11] Schambra UB, Duncan GE, Breese GR, Fornaretto MG, Caron MG, et al (1994) Ontogeny of D1A and D2 dopamine receptor subtypes in rat brain using *in situ* hybridization and receptor binding. Neurosci 62(1): 65–85. 10.1016/0306-4522(94)90315-8 7816213

[12] Brana C, Caille I, Pellevoisin C, Charron G, Aubert I, et al (1996) Ontogeny of the striatal neurons expressing the D1 dopamine receptor in humans. J Comp Neurol 370(1): 23–34. 8797154 10.1002/(SICI)1096-9861(19960617)370:1<23::AID-CNE3>3.0.CO;2-N

[13] Tomassoni D, Bronzetti E, Cantalamessa F, Mignini F, Ricci A, et al (2002) Postnatal development of dopamine receptor expression in rat peripheral blood lymphocytes. Mech Ageing Dev 123(5): 491–498. 11796134 10.1016/s0047-6374(01)00355-4

[14] Qi Y, Wang JK, McMillian M, Chikaraishi DM (1997) Characterization of a CNS cell line, CAD, in which morphological differentiation is initiated by serum deprivation. J Neurosci 17(4): 1217–1225. 9006967 10.1523/JNEUROSCI.17-04-01217.1997 PMC6793738

[15] Wang WC, Juan AH, Panebra A, Liggett SB (2011) MicroRNA let-7 establishes expression of beta2-adrenergic receptors and dynamically down-regulates agonist-promoted down-regulation. Proc Natl Acad Sci U S A 108(15): 6246–6251. 21447718 10.1073/pnas.1101439108 PMC3076830

[16] Heyn J, Ledderose C, Hinske LC, Limbeck E, Möhnle P, et al (2012) Adenosine A2A receptor upregulation in human PMNs is controlled by miRNA-214, miRNA-15, and miRNA-16. Shock 37(2): 156–163. 22249219 10.1097/SHK.0b013e31823f16bc

[17] Jensen KP, Covault J, Conner TS, Tennen H, Kranzler HR, et al (2009) A common polymorphism in serotonin receptor 1B mRNA moderates regulation by miR-96 and associates with aggressive human behaviors. Mol Psychiatry 14(4): 381–389. 18283276 10.1038/mp.2008.15 PMC3162374

[18] Adams BD, Furneaux H, White BA (2007) The micro-ribonucleic acid (miRNA) miR-206 targets the human estrogen receptor-alpha (ERalpha) and represses ERalpha messenger RNA and protein expression in breast cancer cell lines. Mol Endocrinol 21(5): 1132–1147. 17312270 10.1210/me.2007-0022

[19] Huang W, Li MD (2009) Differential allelic expression of dopamine D1 receptor gene(DRD1) is modulated by microRNA miR-504. Biol Psychiatry 65(8): 702–705. 19135651 10.1016/j.biopsych.2008.11.024 PMC2678413

[20] Sun Y, Varambally S, Maher CA, Cao Q, Chockley P, et al (2011) Targeting of microRNA-142–3p in dendritic cells regulates endotoxin-induced mortality. Blood 117(23): 6172–6183. 21474672 10.1182/blood-2010-12-325647 PMC3122940

[21] Potvin S, Stip E, Sepehry AA, Gendron A, Bah R, et al (2008) Inflammatory cytokine alterations in schizophrenia: a systematic quantitative review. Biol Psychiatry 63(8): 801–808. 18005941 10.1016/j.biopsych.2007.09.024

[22] Zalcman S, Murray L, Dyck DG, Greenberg AH, Nance DM (1998) Interleukin-2 and -6 induce behavioral-activating effects in mice. Brain Res 811: 111–121. 9804916 10.1016/s0006-8993(98)00904-4

[23] Zalcman S, Savina I, Wise RA (1999) Interleukin-6 increases sensitivity to the locomotor-stimulating effects of amphetamine in rats. Brain Res 847(2): 276–283. 10575098 10.1016/s0006-8993(99)02063-6

[24] Zalcman S, Green-Johnson JM, Murray L, Nance DM, Dyck D, et al (1994) Cytokine-specific central monoamine alterations induced by interleukin-1, -2 and -6. Brain Res 643(1–2): 40–49. 7518332 10.1016/0006-8993(94)90006-x

[25] Nakano K, Higashi T, Hashimoto K, Takagi R, Tanaka Y, et al (2008) Antagonizing dopamine D1-like receptor inhibits Th17 cell differentiation: preventive and therapeutic effects on experimental autoimmune encephalomyelitis. Biochem Biophys Res Commun 373: 286–291. 18558081 10.1016/j.bbrc.2008.06.012

[26] Nakano K, Yamaoka K, Hanami K, Saito K, Sasaguri Y, et al (2011) Dopamine induces IL-6-dependent IL-17production via D1-like receptor on CD4 naive T cells and D1-like receptor antagonist SCH-23390 inhibits cartilage destruction in a human rheumatoid arthritis/SCID mouse chimera model. J Immunol 186(6): 3745–3752. 21307293 10.4049/jimmunol.1002475

[27] Nakano K, Higashi T, Takagi R, Hashimoto K, Tanaka Y, et al (2009) Dopamine released by dendritic cells polarizes Th2 differentiation. Int Immunol 21(6): 645–654. 19332443 10.1093/intimm/dxp033

[28] Kipnis J, Cardon M, Avidan H, Lewitus GM, Mordechay S, et al (2004) Dopamine, through the extracellular signal-regulated kinase pathway, down regulates CD4+CD25+ regulatory T-cell activity: implications for neurodegeneration. J Neurosci 24(27): 6133–6143. 15240805 10.1523/JNEUROSCI.0600-04.2004 PMC6729670

[29] Eipper-Mains JE, Kiraly DD, Palakodeti D, Mains RE, Eipper BA, et al (2011) microRNA-Seq reveals cocaine-regulated expression of striatal microRNAs. RNA 17(8): 1529–1543. 21708909 10.1261/rna.2775511 PMC3153976

[30] Karpa KD, Lidow MS, Pickering MT, Levenson R, Bergson C (1999) N-linked glycosylation is required for plasma membrane localization of D5, but not D1 dopamine receptors in transfected mammalian cells. Mol Pharmacol 56(5): 1071–1078. 10531415 10.1124/mol.56.5.1071

[31] Bermak JC, Li M, Bullock C, Zhou QY (2001) Regulation of transport of the dopamine D1 receptor by a new membrane-associated ER protein. Nat Cell Biol 3(5): 492–498. 11331877 10.1038/35074561

