# Supplementary figures and images for: MicroRNA 142-3p Mediates Post-Transcriptional Regulation of D1 Dopamine Receptor Expression
Source: PLoS One. 2012 Nov 12;7(11):e49288. doi: 10.1371/journal.pone.0049288 (PMC3495858; doi:10.1371/journal.pone.0049288)

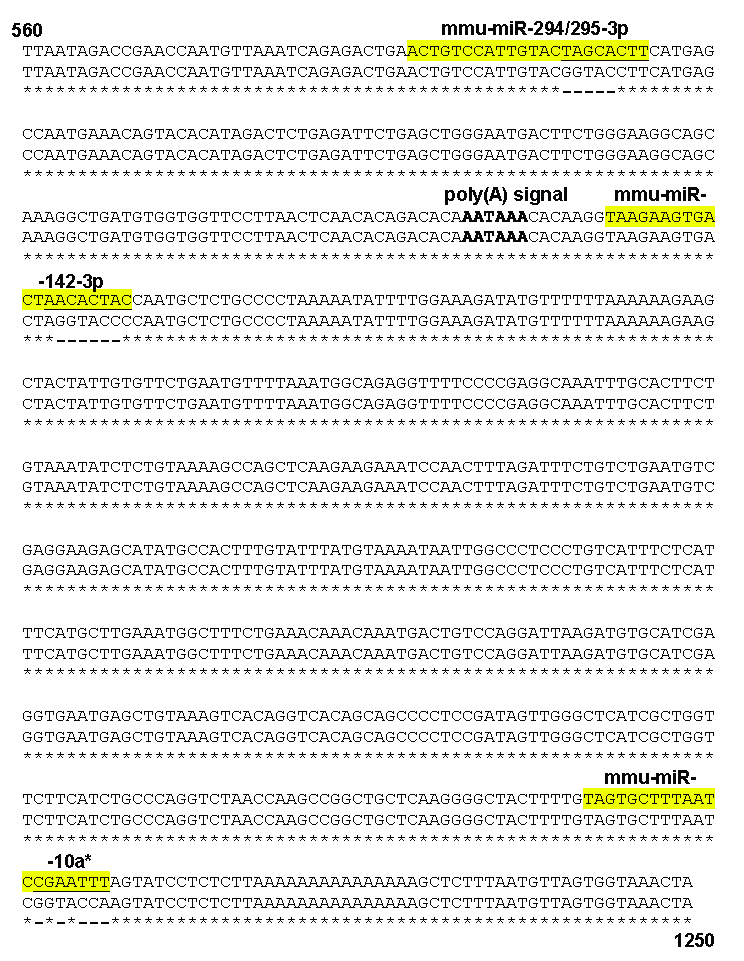

Supplement: Figure S1 — Schematic representation of the binding sites for miR-294/295-3p, miR-142-3p and miR-10a* in D1 receptor 3′UTR and the seed recognition nucleotides that were targeted for mutation. Partial nucleotide sequence (from nucleotides 560 to 1250) of the 1277 bp D1 3′UTR that shows the putative binding sites (highlighted in yellow) for miR-294/295-3p, miR-142-3p and miR-10a*. The poly A signal site is indicated in bold letters. The top strand represents the wild type sequence and the bottom strand is the mutated version. The specific nucleotides that were mutated within each seed recognition sequence (underlined) are represented by a dash. Conserved nucleotides are represented by a *. (TIF) [file pone.0049288.s001.tif]

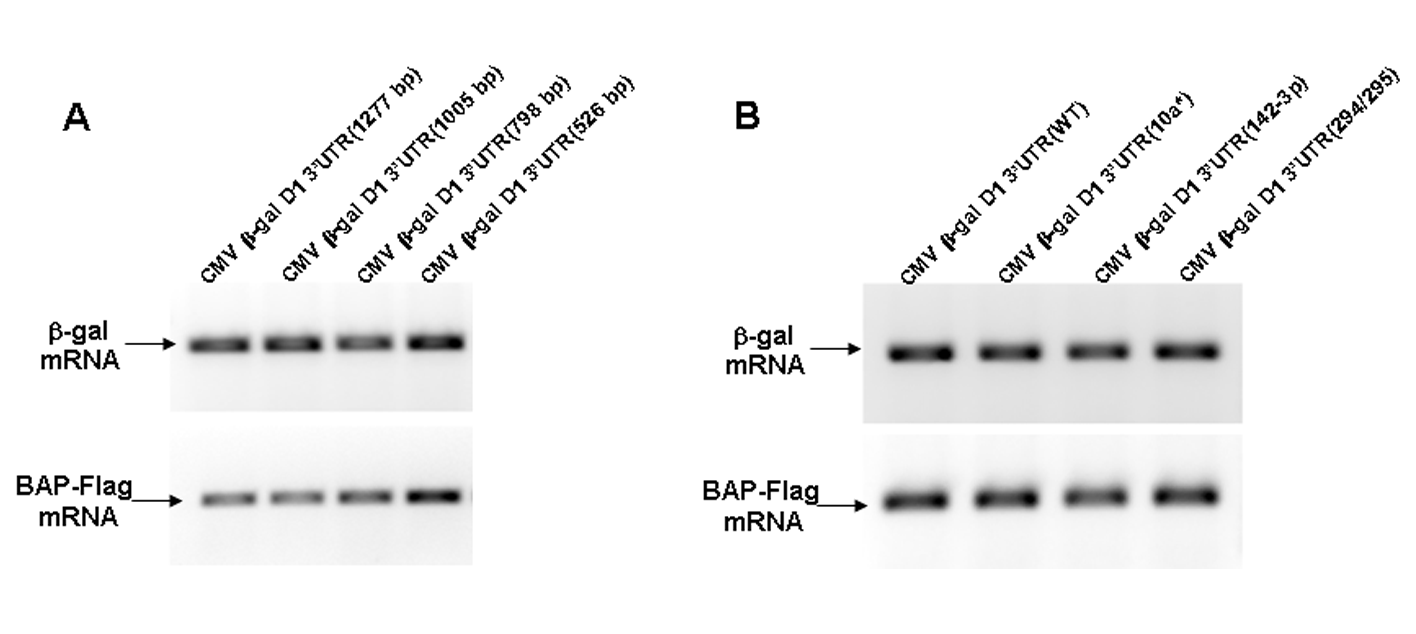

Supplement: Figure S4 — The steady-state levels of β-galactosidase reporter mRNA from the D1 receptor 3′UTR constructs. Constructs with deletions (A) or mutations in the microRNA binding sites (B) is not significantly different. The various reporter constructs were transiently transfected into non-differentiated CAD cells along with a control BAP-Flag™ construct for monitoring transfection efficiency. The mRNA levels were assayed using real-time RT-PCR with the SYBR® green method and primers specific for β-galactosidase and BAP-Flag™. The reactions were stopped in the exponential phase and the products run out on an ethidium bromide stained agarose gel. (TIF) [file pone.0049288.s004.tif]

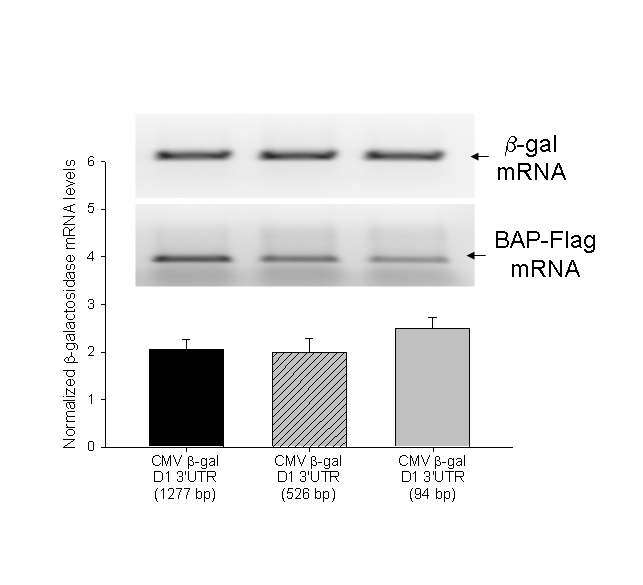

Supplement: Figure S5 — The steady-state levels of β-galactosidase reporter mRNA from the D1 receptor 3′UTR deletion constructs. Cumulative results comparing β-galactosidase mRNA levels between non-differentiated CAD cells transfected with full length D1 3′UTR [pCMV β-gal+D1 3′UTR (1277 bp)] or D1 3′UTR constructs with deletions [pCMV β-gal+D1 3′UTR (526 bp) and pCMV β-gal+D1 3′UTR (94 bp)]. Constructs with deletions were not significantly different. The various reporter constructs were transiently transfected into non-differentiated CAD cells along with a control BAP-Flag™ construct for monitoring transfection efficiency. The mRNA levels were assayed using real-time RT-PCR with the SYBR® green method and primers specific for β-galactosidase and BAP-Flag™. The reactions were stopped in the exponential phase and the products run out on an ethidium bromide stained agarose gel (representative gel is shown in the inset). The bars represent the mean values ± s.e.m. (n = 3). Not significant, P>0.05, One Way ANOVA. (TIF) [file pone.0049288.s005.tif]
